# Operating Theatre Nurse Students' Experiences of the Perioperative Dialogue in Clinical Practice: A Hermeneutical Study

**DOI:** 10.1111/scs.70223

**Published:** 2026-03-19

**Authors:** Ann‐Catrin Blomberg, Lillemor Lindwall

**Affiliations:** ^1^ Faculty of Health, Science and Technology, Department of Health Sciences Karlstad University Karlstad Sweden; ^2^ Lovisenberg Diaconal University College Oslo Norway

**Keywords:** continuity, hermeneutics, operating theatre nurse student, patient safety, perioperative caring, perioperative dialogue

## Abstract

**Background:**

In perioperative care, patient encounters are limited and care planning is not always based on the patient's needs and wishes. Operating theatre nurse students were given the opportunity to use the perioperative dialogue in patient care together with their supervisor. It is of interest what students experience using the perioperative dialogue in clinical practice.

**Aim:**

This study aims to describe the operating theatre nurse students' experiences and reflections of the perioperative dialogues with patients in clinical practice.

**Methods:**

A qualitative approach was chosen and has a hermeneutical design. During clinical practice, the operating theatre nurse students carried out the perioperative dialogues with patients, together with an operating theatre nurse supervisor. Data were text from 116 student examination tasks in a perioperative nursing universal course. This text was interpretive with hermeneutical text interpretation with five steps.

**Results:**

The result showed that the perioperative dialogue is understood as a unique caring process and continuity in the perioperative dialogue created a coherent whole. It offered the opportunity to meet the patient as a unique person, to gain knowledge about them and carry out their perioperative care. Continuity created a mutual community through the care relationship between the patient and operating theatre nurse students. Through the perioperative dialogues, the patient became participated in perioperative nursing care.

**Conclusion:**

The perioperative dialogue serves the patient's desire for dignified care and thereby ensures the provision of higher quality care. It also offers the operating theatre nurse students a new way of caring and arouses reflections about their main task of caring for the suffering patient. The perioperative dialogue with patients offers the possibility to ensure good and safe perioperative care.

## 1 Introduction

This study presents Operating Theatre Nurse (OTN) students' experiences and reflections of perioperative nursing and the perioperative dialogues with patients in clinical practice. The OTN programme takes 1 year at the university and includes both theory and practice of perioperative nursing and the perioperative dialogue. During the OTN students' clinical practice, they carried out perioperative dialogues with the patients they were responsible for together with their supervisor. All students were examined in a seminar where they described the nursing process from their perioperative dialogues with a patient. These dialogues are part of the perioperative caring [1]. The examination contained the OTNs' planning, implementation, and evaluation as well as documentation of the patient's perioperative nursing process. There are studies which focus on the value of perioperative nursing care for patients and the opportunities for OTNs to get to know the unique patient as a person during surgery [2, 3, 4]. However, no studies have been found on OTN students' experiences and reflections from examinations with the perioperative dialogue.

## 2 Theoretical Background

The concept of perioperative nursing practice was developed in the USA in the late 1960s, which replaced the term operating room nursing [5]. The motive was that the OTNs' professional function was considered to be medical technician oriented and that their care did not include the patient's pre‐, intra‐ and postoperative phases [5]. A first definition of perioperative nursing was made by the Association of Perioperative Registered Nurses (AORN) statement committee [6], which described a nursing process thinking within perioperative caring. Additional definitions have been implemented over the years to change the approach from task‐centred to patient‐centred with the justification that care should be based on the patient's problems and needs [7].

In the Nordic countries, the nursing process was developed from a humanistic perspective [8]. ‘Peri’ refers to the time surrounding a patient's surgery and the place where the care takes place [9]. According to Eriksson's caring theory [10] a human being is fundamentally an entity of body, soul and spirit and the body is understood to be a vessel for both health and suffering an abode for human dignity and vulnerability. The caritative caring process as a method can be used to combine science and reality in practice. The caring process includes the following phases: patient analysis, prioritisation of caring, choice of caring actions and activities at different levels of caring and evaluation of caring. The specialist nurse meets and talks with the awake patient and together they plan the patient's care. After surgery the nurse evaluates the perioperative nursing care together with patient to gain more knowledge about what student's experience based on the approach of the perioperative dialogue, we have to reflect on what happens in the unique perioperative nursing process between patient and OTN student.

## 3 The Perioperative Dialogue

The perioperative dialogue was developed in Sweden as an ideal working model for organising perioperative caring from a caring science perspective [9]. Lindwall and von Post [1, 11] describe the perioperative dialogue and emphasise that it should be regarded as a new interpretation of the perioperative nursing process, which includes nursing care and specialised care of the patient. Initially the perioperative dialogue was defined as “Perioperative nursing consists of those nursing actions and nursing activities which are performed by a nurse anaesthetist or operating theatre nurse in pre‐, intra‐ and postoperative phases of the patient's surgery” [9]. Since this definition, several definitions followed.

The latest [3] states “Perioperative nursing is a nurse anaesthetist's and operating theatre nurses' pre‐, intra‐ and postoperative care for a patient who is undergoing surgery.” Perioperative nursing includes all nursing activities related to the surgical treatment, organisation and leadership of perioperative practice. Perioperative dialogues are nurse anaesthetist's and operating theatre nurses' pre‐, intra‐ and postoperative dialogues with the patient, with the purpose to plan, implement and evaluate perioperative nursing care and create continuity.

Previous research from Rudolfsson [12] shows that perioperative dialogues created continuity when the nurse created time for the patient. When the unique patients are given the opportunity to talk to a nurse in the perioperative dialogue about their needs and problems and how they feel and think, their suffering is alleviated. Lindwall [13] described that the patient felt in safe hands and could hand over the control of their body to their nurse anaesthetist or OTN. Lindberg [14] showed that children with special needs who participated in the perioperative dialogue were listened to and prepared for the surgery. The dignity of the children was preserved, and a mutual community was created. Pulkkinen [15] argues that the perioperative dialogue reduces a patient's anxieties and fears related to knee and hip surgery. Other studies show that pre‐ and postoperative conversations with the patient can lead to a better understanding of the patient's health situation [16]. Arvelos Mendes and Clemence Ferrito [2] show that preoperative nursing consultations are important for patients undergoing surgical treatment. Hence the interest in investigating how OTN students experience and reflect on using the perioperative dialogue to plan the patient's perioperative caring.

The aim of the study was to describe the OTNs' experiences and reflections of the perioperative dialogues with patients in clinical practice.

– What are the experiences and reflections the OTN students had after the perioperative dialogues with a patient who had undergone surgery?

## 4 Method

In this study a qualitative approach was chosen. A hermeneutical design based on Gadamer's [17] philosophy of understanding, gained through interpretation was used to gain an understanding of what students experienced in their perioperative dialogues with patients. Gadamer focuses on the concept of pre‐judgment, pre‐understanding and fusion of horizons. Gadamer also emphasizes that those who express themselves and those who understand are connected by a common human consciousness that makes understanding possible [17].

The participants were from a university in the middle of Sweden. The students were registered at a specialist nursing programme in operating theatre care. The training programme collaborates with seven regions, each of which constitutes a study location with 4–8 students. At each study location, there is a main supervisor who is an active OTN.

The specialist nursing programme includes theory and 18 weeks of clinical practice, where the students complete three study assignments. Here, the perioperative dialogues with patients are used to plan, carry out, evaluate, and document the patient's perioperative nursing process. The students present their study assignment at a group meeting led by the main supervisor at the place of study. There is an in‐depth examination task, where the students describe how they have planned the patient's care based on different nursing levels. The documentation includes the perioperative nursing process and also describes experiences and reflects on the perioperative dialogue together with a patient. The examination assignment includes a question answered in writing: *Reflect on experiences of using* “*the perioperative dialogue in clinical practice*”.

Data in this study were from the OTN student's examination between 2017 and 2022. During these 5 years we, as researchers, want to know how students experience implementing theory and to be involved in patients' perioperative nursing in clinical practice. Data include 116 narratives of between four lines to half an A4 page. All students gave their written consent and received information about the aim and method of the study from the responsibility authors of this study.

## 5 Hermeneutic Text Interpretation

Hermeneutic text interpretation was selected to understand the substance of the text before declaring the speaker behind the text. Gadamer [17] highlights the meaning of language for creating the world where reality can be interpreted. The text should be understood and not become another's own interpretation. The understanding of the text is based on the reader's existential and professional pre‐understanding [18]. Therefore, both authors read and interpreted the text. The authors' pre‐understanding consists of the caring science perspective, medical knowledge, values, prejudices, and ethical understanding, as well as our experience as nurse anaesthetist and OTN [18]. The students' answers to the question: *Reflect on experiences of using the “perioperative dialogue”*. Interpretation of the text was initiated with a review of the text as the original source and its relevance in perioperative clinical practice [18].

The text interpretation was done in five steps [18]: *The first reading—integrating the text with the reader*: we started with an open reading, which meant that the first author asked the text: *What experiences did the OTN students have of using the perioperative dialogues in clinical practice?* The interpretation of the texts was influenced by a caring science perspective as well as professional preunderstanding as an OTN. The stories were relevant to the context and the text was read from beginning to end with great curiosity, is this what students experience when using the perioperative dialogue in clinical practice? As interpreter we answer *Yes, it is*.

*The Second Reading—Fusion of Horizons*: Gadamer [17] states that dialogues with the texts lead to a fusion of horizons. Here student experiences of the perioperative dialogues with a patient were revealed. The texts were read carefully, which allowed them to present all their otherness and be a part of the reader. Professional pre‐understanding had been considered in relation to content that was unfamiliar and new questions emerged: *What is important to know for the OTN in planning the patient's care?* Gadamer [17] stated that when our horizon of understanding meets another horizon of understanding, a fusion of horizons occurs, which alters our understanding and a new world opens up for us. In this step, it became apparent that the text had something new to tell.

*The Third Reading—New Questions From the Text*: The following questions arose when the researcher transcended the horizon of the text: *How can the perioperative dialogue influence nursing care within perioperative clinical practice?* The text was read further to find answers to the question, searching for quotations in the text with common and distinguishing qualities, moving back and forth throughout the text for significant expressions.

*The Fourth Reading—Summarising the Main Themes and Subthemes*: The text was carefully read through to search for common features in all significant expressions. The common features were formed into two main themes: each main theme is described separately and is structured using subthemes, which received their design using quotes from the original text.

*The Fifth—A New Understanding*: The whole text was read once again to confirm the themes compared to the text as a whole in search for a new understanding of the whole, from its parts and the parts from the whole which Gadamer describes as the hermeneutic circle. This process of understanding involved abstraction of the main and subthemes to form a coherent text. This step was first done independently by the two researchers. During the process the two researchers with knowledge in perioperative nursing discuss the text, and at a final meeting, consensus of the text interpretations between the two researchers was achieved.

## 6 Ethical Consideration

The principles of research ethics were taken into account in accordance with the Declaration of Helsinki [19], which protects the research subjects ‘anonymity, integrity and maintaining public confidentiality. The students were asked after the examination at the university by the first author, who was the responsibly teacher, whether data could be used for research purposes and they gave their informed consent. Their experiences and reflections were not a part of their final examination. All students’ participation was voluntary. The collected data was treated confidentially and no unauthorised persons had access to the material. Only researchers had access to data, and the data was locked away at the university. The OTN students' quotations are presented in such a way that none of the participants are at risk of being identified [20].

## 7 Results

The result shows experiences and reflections of the perioperative dialogues in clinical practice as two main themes: The perioperative dialogue is understood as a unique caring process and the continuity of the perioperative dialogue creates a coherent whole.

The perioperative dialogue is understood as a unique caring process consisting of three subthemes*: to meet the patient as a unique person, to get knowledge about the patient*, and *to carry out the patient's perioperative care*. Continuity in the perioperative dialogue creates a coherent whole consisting of two subthemes: *mutual community; the patient becomes involved in their perioperative care*.

### 7.1 To Meet the Patient as a Unique Person

The students believe that by meeting the patient in pre‐, intra‐ and postoperative dialogues, it is possible to get to know the patient as a unique person and not just as a surgery on an operating list. It is important to get eye contact with the patient and to listen to what the patient wants to say and show the patient that there is time for reflection.

A student writes: *the main purpose of the conversations was to meet the patient and thus get to know the patient and get an overall picture. It is easier to see the patient as more than a procedure when I get an overall picture of who the patient is* [3].

### 7.2 To Get Knowledge About the Patient

The preoperative dialogue contributes to the student acquiring knowledge through the patient's story, which could not be obtained through the operation report or the patient's medical record. The additional information contributed to the student gaining a deeper understanding of the patient and the planned surgery. Through the perioperative dialogue, new knowledge about the patient became important for the surgical team's planning. This new knowledge also meant less stress and a more effective collaboration between the professions in the surgical team.

A student says*: I need to talk to the patient to supplement the operation report and medical records with “primary information”. Through the conversation, the patient can provide information that is both current or not included in the medical record at all. In addition, the patient can tell us what they think should be prioritised right now, as the patient and the staff may have different opinions about the matter* [2].

Another student experienced: *Thanks to the perioperative dialogue, relevant measures could be planned in time. Important goals for the patient could be set. Stress was minimal intraoperatively thanks to the preparation based on the patient's needs and the care flowed calmly yet efficiently; everyone in the surgical team was “on board” thanks to information that emerged in the preoperative conversation being shared with the other participants* [3].

### 7.3 To Carry Out the Patient's Perioperative Care

There was an opportunity to plan the patient's nursing in consultation with the patient and based on the patient's problems and needs, which gave a clearer structure. The student gains an increased knowledge of how injuries can be prevented and how the patient's resources can be utilised. In the perioperative dialogue, the choice of care actions and care activities is prioritised based on the patient's problems and needs related to the surgical treatment and time is given to alleviate the patient's suffering. The patient becomes involved in their care and confirmed as a person in a high‐tech environment. Students experienced that some patients showed gratitude postoperatively, which was a positive experience and a satisfying feeling. The conversations contributed to the patient's unique care process and a more efficient collaboration in the surgical team.

A student writes: *The planning flows more easily and it goes faster inside the room, I think, when you know in advance what to expect. I am responsible for the patient; I can anticipate risks and mitigate/prevent them with the help of the team and relevant care measures* [6].

Another student writes: *To base nursing needs on the basis of conversations with the patient and their story and to be able to map health status, well‐being and what resources are available… necessary to be able to alleviate, encourage, show understanding and empathy as well as confirm any worries and fears…. I understood that the patient in my case expressed gratitude and relief when he was discharged* [4].

The results show how important it is to be able to reflect based on the perioperative nursing process. Through the perioperative dialogue, the student is given the opportunity to meet and get to know the unique patient. Knowledge was acquired from the patient through pre‐, intra‐ and postoperative conversations and these contributed to the student getting an overall picture of the patient's problems and situation. The student gained a deeper understanding of the patient's needs and wishes, which is important to ensure the patient's perioperative nursing care. The ability to carry out the perioperative dialogue with the patient helps to create a sense of trust and that the OTN cares about the patient in a high‐tech environment. The perioperative dialogue provides the student with a reflection model where they can structure the care work and streamline collaboration in the surgical team.

### 7.4 Mutual Community

The students experienced that through the perioperative dialogue they gained a relationship with the patient and created a mutual community. This was not created for patients when the preoperative meeting took place only in the operating room. Bodily touch enhanced the feeling of trust and that could lead to an in‐depth conversation. The care relationship between patient and caregiver contributed to the experience of being more prepared and able to give the patient dignified care. The students believe that future colleagues will want the opportunity to create a mutual community for the patient. In the postoperative conversation and in the evaluation of completed care, students received patients' appreciation.

A student writes*: It is an important task as OTN to build a relationship with the patient so that they can feel safe during surgery when the patient is physically exposed. I have noticed that I have a different relationship with the patients I have met pre‐, intra*, *and postoperatively, and I carry those patients with me in a different way than those I only meet in the operating room* [3].

Another student explains*: Touching the patient, which can easily be expressed as taking his or her hand or placing a hand on the shoulder, can also strengthen the feeling of trust and security in the care and pave the way for a deeper conversation* [1].

### 7.5 The Patient Becomes Involved in Their Perioperative Care

The perioperative dialogue gave the student the opportunity to involve the patient in caring. The participation led to a genuine commitment between caregiver and patient, which made the patient dare to “open up”, to get answers to unanswered questions. This meant that the patient gained confidence in the caregivers, which contributed to the patient's surrender into safe hands.

A student writes: *It is extremely important to feel empathy for the patient for the conversation to feel “genuine” to him/her. A patient can easily sense whether there is a genuine commitment on your part* [1].

Another student writes*: By gaining the patient's trust, you can try to steer the conversation to get the patient to open up. In this way, the patient becomes involved and I can respond and try to answer the questions that the patient may not otherwise have dared to ask, in the best way and to try to instil a sense of security and calm in him* [1].

When OTN students conduct the perioperative dialogue, they gain a mutual community with the patient. In a mutual community, there is trust and a caring relationship. The patient felt involved in their perioperative nursing care when they were able to talk about the surgical procedure. Future OTN colleagues said that the patient was well prepared for how the care would be planned and implemented. Students felt that they could maintain the patient's dignity and the treatment relationship ended in a dignified manner when the patient showed their appreciation.

## 8 The New Understanding

The results led to a new understanding of OTN students' experiences and reflections on the perioperative dialogue in clinical practice. When using the perioperative dialogue, OTN students show the patient that he/she is interested, are happy to share their knowledge, and that they can ask their questions. In the perioperative dialogue, the caring relationship appears to be important, a different relationship than when the meeting with the patient first takes place in the operating room.

The mutual community leads to the patient feeling safe; it enhances trust in the caregiver and means the patient becomes more involved in their care. The result makes us aware that the perioperative dialogue gives patients who are about to undergo a surgical treatment the opportunity to tell their story and caregivers who see and listen and plan the care based on their problems and needs. The interpretive module shows a new way of structuring the patient's perioperative nursing care when the perioperative dialogue is understood as a unique treatment process. The perioperative dialogue gives meaning as the student will be by the patient's side throughout the perioperative nursing process. Continuity in the perioperative dialogue creates a coherent whole, which is a prerequisite for the nurse and the patient to be part of a mutual community, for true authenticity and for an invitation to participate in their care to take place. One possibility to create a coherent whole is to organizationally highlight the need to have the opportunity to obtain the patient's story, important knowledge that can be important for the planning of the patient's perioperative care. The interpretation shows that continuity in the patient's perioperative care provides opportunities to create a care relationship where nurses have the responsibility not to abandon the patient in a vulnerable situation.

## 9 Discussion

The study's result presented experiences and reflections from the OTN students' regarding the use of the perioperative dialogue with patients in clinical practice. The result shows that the students experience and reflect that the perioperative dialogues enabled them to care for the patients based on a unique perioperative nursing process, that work became more structured and the caring part of a unique nursing process. All students in the study described that it was positive to be able to carry out the perioperative dialogue. It was revealed in the OTN students' reflections that they have a desire to have the perioperative dialogue as a working model in the future. The model can help to structure patient perioperative nursing care and the approach achieves something of value for both OTN and the patient. Through the perioperative dialogue, new information emerged when OTN students were given access to the patients' story, which according to Ekman et al. [21] captures the person's suffering as opposed to the medical narrative and is the starting point for person‐centred care.

In OTN education, the student learns Erikssons´ caritative caring theory and this perspective was the foundation of the development of the ideal working model the perioperative dialogue. Before clinical practice students had a theory course in perioperative nursing and perioperative dialogues. Here students have the opportunity to get to know the patient as a unique person, as body, soul and spirit, and to gain knowledge to be able to carry out the patient's care in connection with a surgical treatment. It was also about acquiring knowledge about the patient's background and situation as well as the patient's medical records and organising their time for a meeting with the patient. According to Blomberg et al. [22], the OTNs got to know the patient as a unique person and created continuity in the patient's care. The students were there when it happens and a part of the clinical situation. There is an opportunity in the perioperative nursing process to see, listen and feel what is happening in the meeting and understand which problems and needs should be prioritised for the patient and avoid the care being carried out routinely. OTN students believe that by using the perioperative dialogue, they were able to create a care relationship and make the patient involved in their care. Hanssen et al. [23] argue that listening to the patient is an important part of planning the patient's care, but it is difficult to free up time in perioperative care because the demand for productivity is high. The meeting is usually short and requires the OTNs to carry out a conversation that leads to a relationship. Usually, this conversation takes place in connection with the transport into the operating room. Martinsen [24] writes that if the caregivers want the person's story, trust is required: There is a difference between seeing and perceiving the other and registering or classifying the other person from a distance. Blomberg et al. [3, 25] showed that OTN's try to prioritise a preoperative meeting, partly because they want to meet the patient face to face and confirm their presence. It also meant that OTN's took more responsibility and protected the patient's body from being exposed to injury and preserved the patient's dignity. The importance of students having time to obtain preoperative information about the patient here was confirmed by Eriksson et al. [26].

In a high‐tech environment, there is a risk that the focus shifts from nursing care towards technology and medicine, which hinders the development of person‐centred care [27]. Newly graduated nurses highlighted that caring in perioperative practice was more task‐oriented than focusing on the patient as a person and the different care required than from the general ward [26].

Suganandam [28] described that new information also communicates with the surgical team any possible care alteration. The goals and quality of care can be improved by gaining knowledge from the patient. The students have an explicit desire to create a caring relationship with their patient. Future colleagues agree that the model contributes to better planning and implementation of the patient's care in connection with surgery. Only having short conversations with the patient inside an operating room does not lead to increased patient safety and the care does not become more efficient. This is confirmed in previous studies [1, 11, 29]. Introducing something new in operating theatre care does not necessarily mean more productivity or efficiency, but initiating improvement work such as introducing the perioperative dialogue should lead to increased trust and safety from a patient perspective [30]. The responsibility often lies with the leadership to initiate various innovations and OTN students who become active after completing their education should be able to be part of similar innovation projects. Blomberg et al. [31] argue that OTNs were prevented from being there for the patient because perioperative practice is governed by an efficiency and production mindset as well as by habits and routines. This was also shown in Lindwall and von Post's [11] study that there are habits and obstacles to introduce the perioperative dialogue. Eriksson et al. [26] described that OTN's met the patient and asked only the most necessary questions related to the current surgery, but the participants in the study expressed the need for more patient contact and to use the perioperative dialogue. They mean it was difficult to regard the patient as a person and not a surgical procedure. There is a willingness on the part of OTNs according to Blomberg et al. [22] to follow the patient all the way to ensure continuity of care. OTN students reflected that the perioperative dialogue created the opportunity for continuity in the patient's care and that they were the continuity, as they had created a care relationship with the patient. Arakelian et al. [16] also described the importance of continuity of care, to be welcomed by a familiar face and to be able to hand over the responsibility in safe hands, and a dignified end postoperatively. McCormack and McCance [32] argue that central to person‐centred care is to be in a relationship, to be in a social world, to be present and to be with oneself. An end to the care relationship through the postoperative visit will share the experiences according Suganandam [30] of both patient and the nurse and provide an opportunity to evaluate the care provided. The clinical competence that students develop during clinical practice includes ethical valuation skills and an awareness of the power relationship between patient and caregiver. Future studies should focus on power structures within the perioperative context.

## 10 Methodological Consideration

This study has some limitations. The first author carried out the interpretation, and the texts were reviewed together with the second author. Both are researchers in perioperative nursing care, and this may have affected the results of the study, but also the credibility of the study. The number of students' reflections could be a limitation and that they were OTN students from one university in the middle of Sweden, but a strength that they represented seven regions in Sweden. Through hermeneutic text interpretation, the reliability of the study. New knowledge and deepened understanding emerged about OTN students' use of the perioperative dialogue in the planning of the patient's perioperative care, which may be relevant in a clinical setting. Transferability was not present in all findings, but it was positive that the students were interested in implementing the ideal perioperative dialogues in clinical practice to their future way.

## 11 Conclusion

This study has shown that OTN students get a deeper understanding when they have time to develop the perioperative nursing process together with the patient, because the perioperative dialogues offer the students a new way of caring and arouse experience and reflections about their main task to care for the suffering patient. Through knowledge and reflection on the results of the study, the future OTN should take responsibility for meeting the patient who is going to undergo surgery before, during and after the operation for the development of person‐centred care. What is needed is that organisations have a clear ambition and will to change by taking part in research in perioperative nursing care. A responsible project manager is appointed, who can both provide education in the perioperative dialogue and who clarifies for the healthcare organisation the importance of having a systematic implementation work under scientific guidance in clinical practice. The future OTN should have solid scientific and evidence‐based education and can, with increased ambitions, ensure good and safe perioperative care. More research is needed to develop the theory and clinical practice.

## Author Contributions

A.‐C.B. and L.L. jointly designed the study. All narratives were collected by A.‐C.B. The analysis was led by A.‐C.B. in close collaboration with L.L. A.‐C.B. drafted the manuscript, and both authors critically reviewed and approved of the final version.

## Funding

The authors have nothing to report.

## Conflicts of Interest

The authors declare no conflicts of interest.

## Data Availability

The data that support the findings of this study are available on request from the corresponding author. The data are not publicly available due to privacy or ethical restrictions.
